# Rising demand for intensive care in pediatric pancreatitis without an increase of case numbers or disease severity: a 16-year retrospective single-center cohort study

**DOI:** 10.1186/s40348-026-00265-w

**Published:** 2026-11-10

**Authors:** Friederike Kunkel, Mechthild Wegner, Annegret Winkler, Ali Aghdassi, Hagen Bahlmann, Matthias Heckmann, Jan Baier

**Affiliations:** 1 Department of Neonatology and Pediatric Intensive Care, University Medicine Greifswald, Ferdinand-Sauerbruch- Strasse, Greifswald, 17475 Germany; 2 Department of Internal Medicine A, University Medicine Greifswald, Greifswald, Germany; 3 DZKJ (German Centre for Child and Adolescent Health), Partner Site Greifswald/Rostock, Greifswald, Germany

**Keywords:** Acute, Pancreatitis, Pediatric, Intensive care, Etiology, Severity, Biliary, Obesity

## Abstract

**Background:**

Between May 2024 and November 2025 eight children with pancreatitis were treated at our pediatric intensive care unit, seven of them because of the pancreatitis itself. We reviewed all pediatric pancreatitis admissions to our center from 2010 to 2025 in order to clarify whether a trend of case numbers, etiology or severity explains this clustering.

**Methods:**

All inpatients under 18 years with an ICD-10 code K85–K87 were identified by a search of our central clinical controlling and reviewed based on the INSPPIRE definitions. An episode required lipase or amylase at least three times the contemporaneous upper limit of normal or diagnostic imaging, together with a compatible presentation. Exclusion criteria included, among others, conditions involving exocrine pancreatic insufficiency, such as cystic fibrosis, or patients on follow-up visits. Severity was graded according to the NASPGHAN classification.

**Results:**

Of 184 identified admissions, 59 patients met the inclusion criteria: 44 acute, 7 acute recurrent and 8 chronic pancreatitis. Median age was 12 years (IQR 8–16), median stay 7 days (IQR 3–12). Biliary disease and inflammatory bowel disease were the most frequent etiologies with 10 patients each, followed by trauma and genetic variants with 7 each; 4 remained unexplained. The index episode was mild in 26 patients, moderately severe in 25, severe in 5 and could not be graded in 3. 15 patients were treated at an intensive care unit, in 9 of them the pancreatitis was the leading reason. 7 of these 9 admissions occurred in 2024–2025, one each in 2011 and 2014 and none in the nine years in between. The annual number of treated patients did not increase (10 in 2024–2025 versus 49 before), and the proportion of severe disease did not either (1/10 versus 4/49).

**Conclusions:**

Case numbers and severity distribution remained stable over 16 years while the need for intensive care rose markedly in the last two years. Referral of complicated cases after advanced pediatric endoscopy became available locally in 2020 and a lower admission threshold are more plausible explanations than a change of the disease. A perceived local increase of severe pediatric pancreatitis should be checked against the complete case series of the center before it is reported as an epidemiological observation.

## Background

Acute pancreatitis (AP) in childhood has traditionally been regarded as a rare disease entity [1]. Contemporary population-based data estimate the incidence of pediatric AP at ~ 8/100,000 children annually, with a considerable variation between global regions [2–4]. The etiologic spectrum of pediatric pancreatitis diverges from adults, encompassing biliary disease [3], monogenic variants [3, 5], immune-mediated conditions like inflammatory bowel disease (IBD) [6], metabolic disorders [7], trauma and drug-induced injury [8, 9]. Standardized diagnostic criteria—as established by Morinville et al.—are essential for accurate surveillance and epidemiological comparability [10, 11]. While most cases follow a self-limiting course, severe complications like pancreatic necrosis, SIRS and organ failure do occur [12].

The University Medicine Greifswald is the tertiary referral center for children in the eastern part of the state Mecklenburg-Vorpommern, a rural region at the German Baltic coast with a small resident pediatric population and a large seasonal influx of holidaymakers. Between May 2024 and November 2025 eight children with pancreatitis were treated at our pediatric intensive care unit (PICU), one of them died from infected pancreatic necrosis after biliary acute pancreatitis. In seven of these eight children the pancreatitis itself was the reason for intensive care. Being concerned that recent epidemiological evidence indicates a rising global incidence of pediatric pancreatitis [4, 7, 13, 14] and our region is considered a high prevalence area for obesity and associated diseases [15], these seven admissions were the reason for the present study. We reviewed all pediatric pancreatitis admissions to our institution from 2010 to 2025 with the aim to clarify whether a trend of case numbers, etiology or disease severity over these 16 years explains the recent clustering, or whether it has to be regarded as a chance observation.

## Methods

### Setting and design

This is a retrospective cohort study at the Department of Pediatrics of the University Medicine Greifswald. Pediatric intensive care is provided by a single unit that admits all children except those after transplantation of solid organs. Advanced pancreaticobiliary endoscopy in children is performed together with the Department of Internal Medicine A. The study period comprises the 16 calendar years from 2010 to 2025.

### Case identification and definitions

All inpatients under the age of 18 years with an ICD-10 discharge code K85, K86 or K87 were identified by a database search of our central clinical controlling. Patients with a disease leading to exocrine pancreatic insufficiency without pancreatitis, e.g. cystic fibrosis or Shwachman-Diamond syndrome, and patients coded after pancreatic head resection for congenital hyperinsulinism were excluded. Admissions representing follow-up imaging, elective endoscopic procedures or a later recurrence of a patient already counted were not treated as index admissions. For all remaining patients the complete discharge documentation was reviewed.

Diagnoses were verified based on the INSPPIRE definitions [11]. The laboratory reference interval for serum lipase changed twice during the study period, from 1.59 to 6.36 µkat/L until 2022 to 0.216–1.25 µkat/L in 2024 and 0.2–0.88 µkat/L in 2025 (no patient was included in 2023). Absolute enzyme values are therefore not comparable across the period, and all enzyme results are given as multiples of the upper limit of normal (ULN) valid at the time of measurement, in the text as well as in Table 1. Laboratory results were taken from the discharge documentation and from the hospital laboratory system and refer to the index admission. 

**Table 1:** Children treated at the pediatric intensive care unit during a pancreatitis episode, May 2024 to November 2025 (n = 8); a Highest value within the first 72 hours, expressed as a multiple of the upper limit of normal (ULN) valid at the time of measurement: 1.25 µkat/L in 2024 and 0.88 µkat/L in 2025. b The only patient of this group in whom the pancreatitis was not the leading reason for intensive care; the stay followed percutaneous drainage of post-traumatic pseudocysts. c Serum lipase remained within the reference interval; the diagnosis rested on imaging and on the lipase and amylase content of the aspirated cyst fluid. BMI, body mass index; CRP, C-reactive protein; CT, computed tomography; ERCP, endoscopic retrograde cholangiopancreatography; EUS, endoscopic ultrasound; F, female; M, male; P, percentile; WBC, white blood cell count

Case	Age (y), sex	Etiology		Severity	Lipase (× ULN)^a^	WBC (× 10⁹/L)	CRP (mg/L)	Intervention	Outcome	Stay (d)
1	16, F	Biliary		Mild	40	8.5	41	ERCP with papillotomy, stone extraction and biliary stent; laparoscopic cholecystectomy	Recovery	13
2	11, F	Genetic (SPINK1), acute recurrent		Mild	44	14.6	22	Conservative, intensive care for refractory pain	Recovery, recurrent disease	2
3	13, M	Biliary, severe obesity		Severe	24	41.6	342	ERCP with biliary stent, papillotomy withheld because of coagulopathy; papillotomy and stone extraction at repeat procedure	Death	17
4	12, F	Biliary		Moderately severe	106	13.5	6	ERCP with papillotomy, stone extraction and biliary stent	Recovery	4
5	16, M	Genetic (CTRC), later chronic		Moderately severe	32	18.8	171	EUS-guided transgastric drainage with lumen-apposing metal stent and double-pigtail prostheses	Recovery, recurrent disease	34
6	11, M	Autoimmune-associated (type 1 diabetes)		Moderately severe	7.5	28.7	99	Conservative	Recovery	19
7	2, F	Parainfectious (*Campylobacter*) or drug-related		Moderately severe	40	not documented	38	Conservative	Recovery	3
8^b^	13, F	Trauma (pancreatic laceration with pseudocysts)		Moderately severe	within normal range^c^	13.2	78	CT-guided percutaneous drainage	Recovery	19

An episode of AP was counted if serum lipase or amylase reached at least three times the contemporaneous ULN, or if cross-sectional imaging or endoscopic ultrasound was diagnostic, in each case together with a compatible clinical presentation. Borderline records—an enzyme elevation below the three-fold threshold together with a pancreatitis diagnosis of the treating team—were adjudicated on the full clinical documentation by two of the authors; 2 patients were included on that basis and are identified in the sensitivity analyses. Acute recurrent pancreatitis (ARP) required at least two distinct episodes with complete resolution in between, chronic pancreatitis (CP) required imaging, histological or functional evidence of chronic disease. Patients were classified once, at the highest category reached during the study period, and all analyses are given per patient and not per admission.

Severity of the index episode was graded as mild, moderately severe or severe according to the classification of the North American Society for Pediatric Gastroenterology, Hepatology and Nutrition (NASPGHAN) [16]. Etiology was assigned from the discharge documentation and not from the diagnostic code and recorded as a single leading cause. Where IBD and a drug exposure coincided, both were recorded and the patient is counted under IBD. Obesity was recorded when documented in the discharge letter.

Two definitions of intensive care were applied. The broad definition counts every patient treated at an intensive care unit at any time during a pancreatitis episode, the restrictive definition only those in whom the pancreatitis itself was the leading reason for admission. Intensive care admissions were attributed to the calendar year in which they occurred.

### Analysis

Because of the small number of patients the analysis is mainly descriptive. Continuous variables are given as median with interquartile range (IQR) and range, categorical variables as counts and proportions. The temporal distribution of the intensive care admissions was additionally examined by Poisson regression with the calendar year as a continuous covariate, which requires no choice of a time window, and the probability of the number observed in 2024–2025 under a uniform distribution across the 16 years was derived from the Poisson distribution; given the small number of events these results are exploratory. Two sensitivity analyses repeat the evaluation, once without the 2 adjudicated patients and once with a two-fold instead of a three-fold enzyme threshold. All analyses were performed using Stata 19.5 (Stata Corporation, College Station, TX, USA).

## Results

### Cohort

From 2010 to 2025, 184 pediatric inpatients with an ICD-10 code K85–K87 could be identified in our database. 84 admissions in 19 patients were excluded because the code reflected exocrine pancreatic insufficiency without pancreatitis (75 admissions in 11 patients with cystic fibrosis, 3 admissions in 2 further patients) or the state after pancreatic head resection for congenital hyperinsulinism (6 admissions in 6 patients). A further 25 admissions represented follow-up imaging, elective endoscopic procedures or later recurrences of patients already counted.

**Fig. 1:**
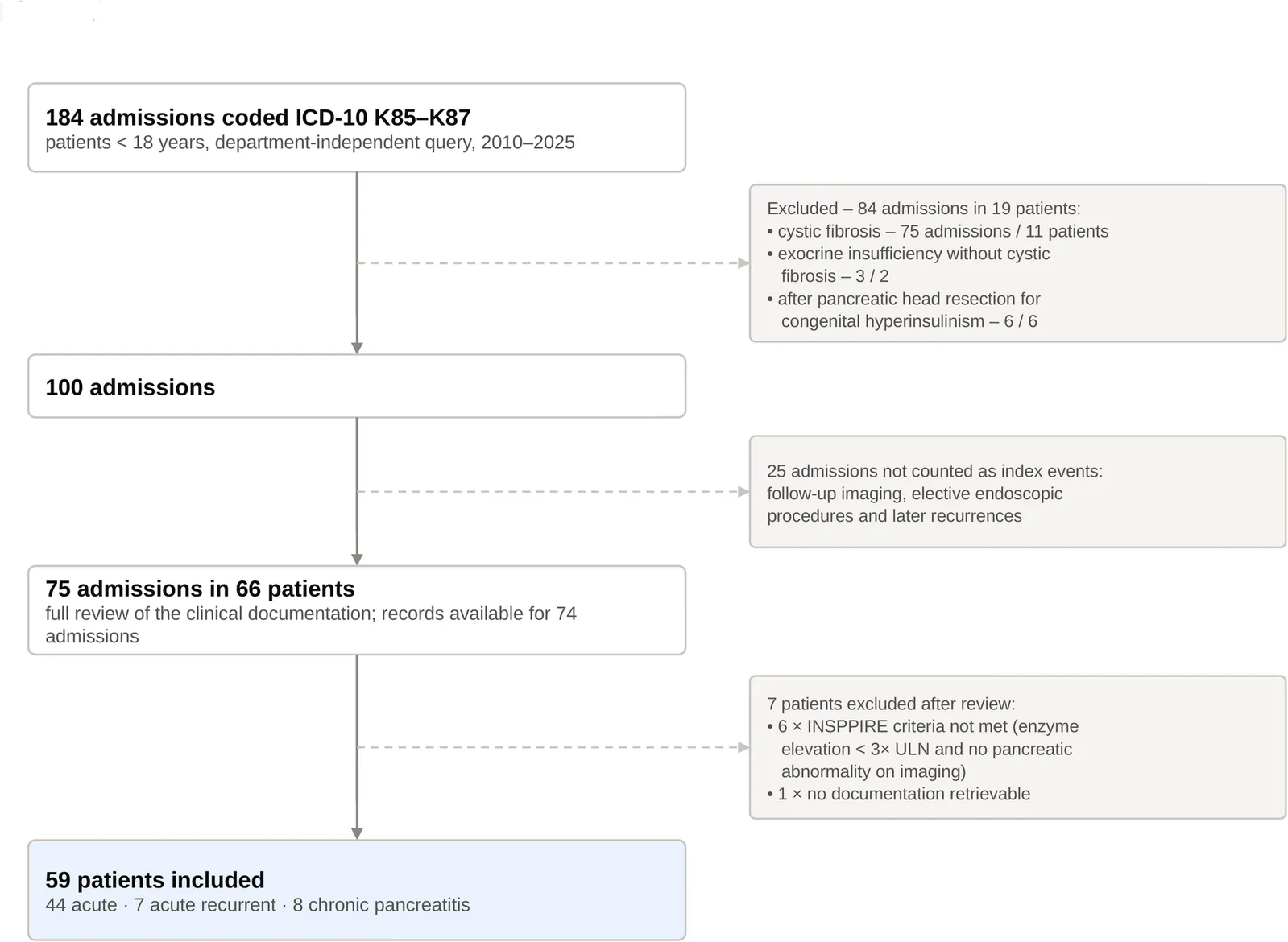
Identification and inclusion of patients, 2010–2025. Admissions of patients under the age of 18 years with an ICD-10 code K85–K87 were identified by a database search of our central clinical controlling and reviewed based on the INSPPIRE definitions. Abbreviations: ULN – upper limit of normal of serum lipase; the reference interval changed twice during the observation period, and the three-fold criterion was applied against the value valid at the time of measurement

The remaining 75 admissions in 66 patients were reviewed in full; discharge documentation was available for 74 of them. 7 patients were excluded after review: in 6 the INSPPIRE criteria were not met, and in 1 patient no clinical documentation could be retrieved. 59 patients formed the analysis cohort (Fig. 1): 44 with AP, 7 with ARP and 8 with CP.

Thirty-three patients were females and 26 males. Median age at the index episode was 12 years (IQR 8–16; range 1–17), median length of the index hospital stay 7 days (IQR 3–12; range 1–40). 9 patients experienced more than one documented episode during the observation period. Obesity or overweight was documented in 13 patients. Cohort characteristics by disease category are shown in Table 2. 

**Table 2:** Characteristics of the cohort by disease category, 2010–2025; IQR, interquartile range

Characteristic	Acute pancreatitis (*n* = 44)	Acute recurrent pancreatitis (*n* = 7)	Chronic pancreatitis (*n* = 8)	All patients (*n* = 59)
Female / male	24 / 20	4 / 3	5 / 3	33 / 26
Age at index episode, years, median (IQR)	13 (9–16)	10 (7.5–13)	9 (7.8–12.8)	12 (8–16)
Length of index stay, days, median (IQR)	8 (3.8–13.3)	6 (3.5–6.5)	4 (2.8–10.5)	7 (3–12)
Severity of index episode				
— mild	18	5	3	26
— moderately severe	19	2	4	25
— severe	5	0	0	5
— not assessable	2	0	1	3
Intensive care during a pancreatitis episode	13	1	1	15
— pancreatitis the leading reason	7	1	1	9
More than one documented episode	0	7	2	9
Obesity or overweight documented	11	2	0	13
Resident outside the catchment area	8	0	4	12

Twelve patients did not live in our catchment area. These were almost exclusively children of holidaymakers who fell ill or were injured while spending their vacation at the Baltic coast.

### Etiology

**Fig. 2:**
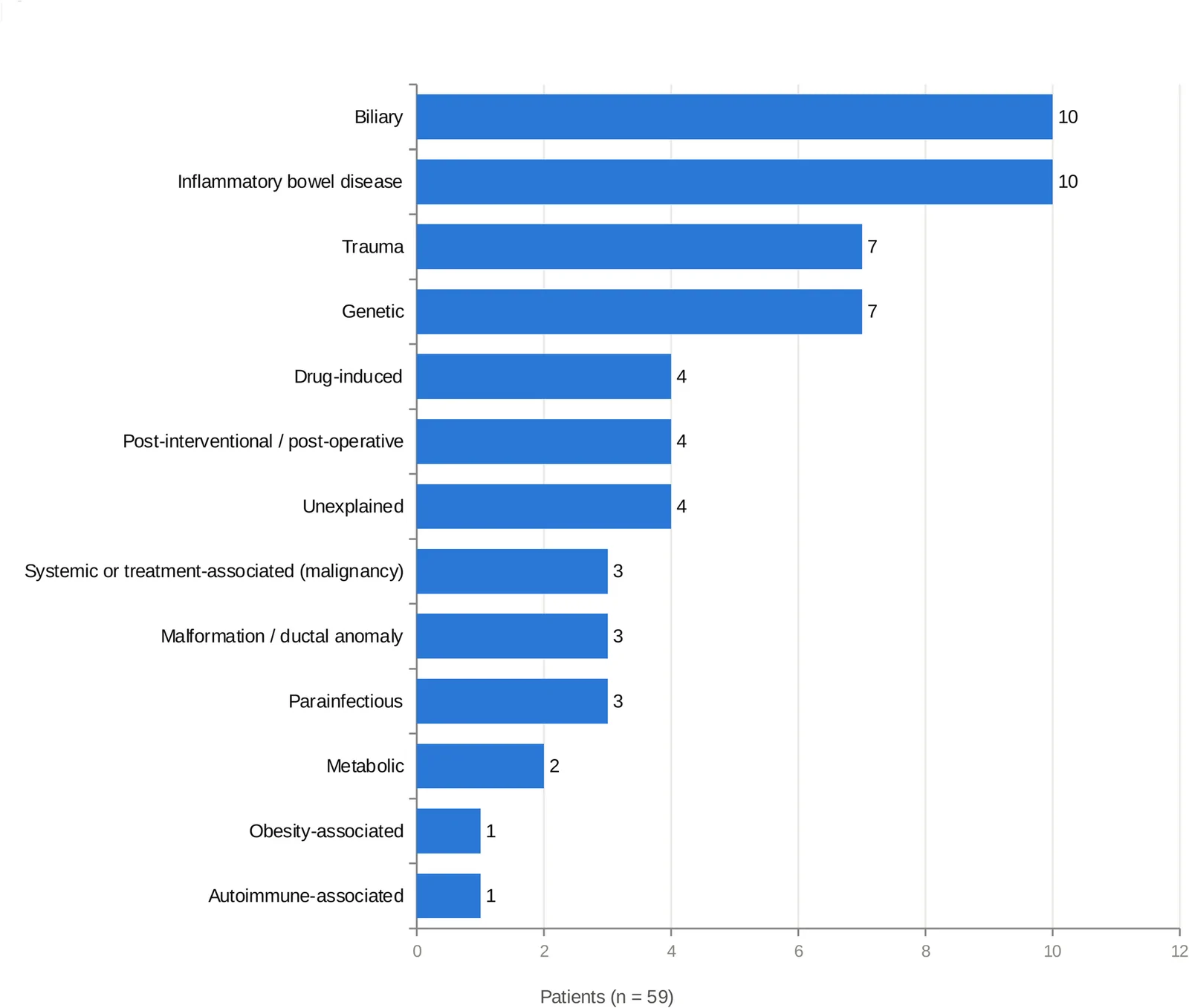
Etiology of pancreatitis at the patient level, years 2010–2025 (*n* = 59). Two of the ten IBD-associated episodes were attributed to a concurrent drug exposure (azathioprine, mesalazine) and are shown in the IBD category

Biliary disease and IBD were the most frequent etiologies with 10 patients each; in 2 of the IBD patients the episode was attributed to a concurrent drug exposure. All further etiologies are shown in Fig. 2. Only 4 of 59 patients remained without an explanation after complete review, and 7/10 patients with biliary pancreatitis were obese or overweight.

### Severity and course

**Fig. 3:**
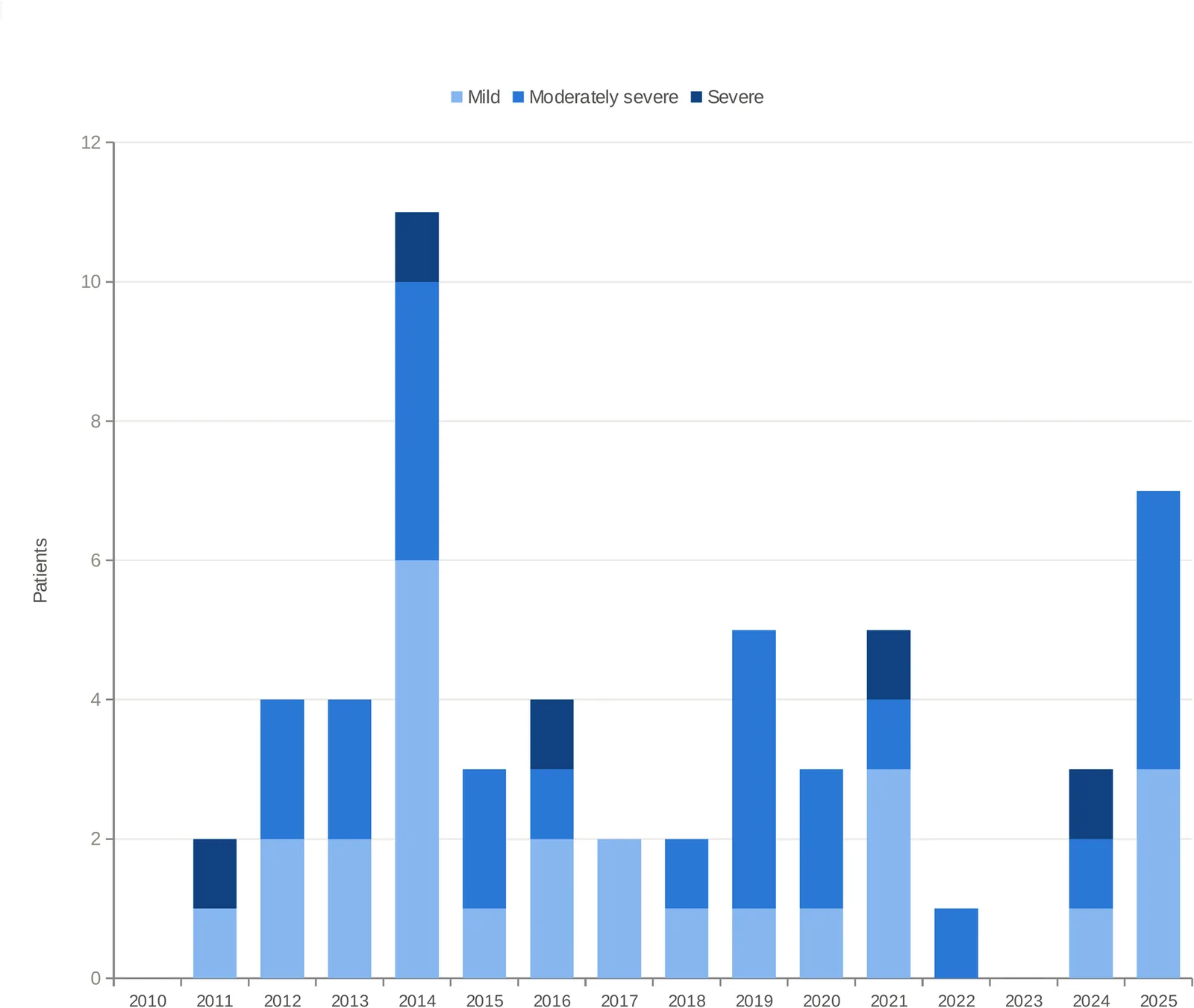
Severity of the index episode according to the NASPGHAN classification, by calendar year of first diagnosis. No patient was included in 2010 and 2023. Three patients whose documentation did not permit grading are not shown

The index episode was mild in 26 patients, moderately severe in 25 and severe in 5; in 3 patients the documentation did not permit grading. The distribution of severity did not shift over the study period (Fig. 3). Patients who required intensive care stayed considerably longer than those who did not (median 17 days, IQR 9–27.5, versus median 6 days, IQR 3–9). One patient died, an adolescent with severe obesity and biliary necrotizing pancreatitis, giving a case fatality of 1 in 59 over 16 years.

### Intensive care over time

Fifteen of the 59 patients were treated at an intensive care unit at some time during a pancreatitis episode. In 9 of them the pancreatitis was the leading reason for intensive care; the remaining 6 were admitted primarily for trauma or its interventional treatment (4), for complications of leukemia therapy (1) or for hepatic veno-occlusive disease during treatment of a stage IV neuroblastoma (1), with pancreatitis as an accompanying diagnosis.

**Fig. 4:**
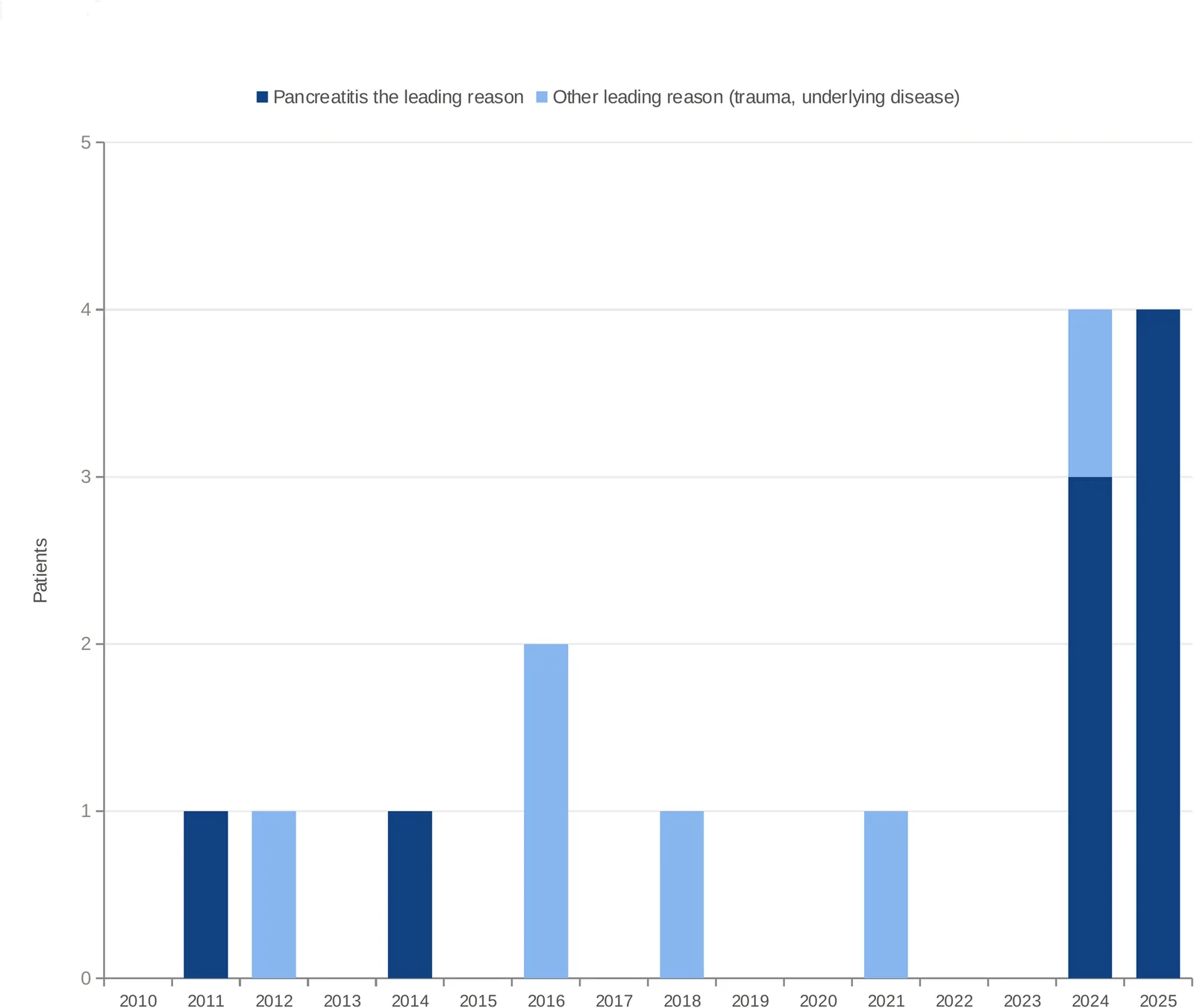
Patients treated at an intensive care unit during a pancreatitis episode, by calendar year of the intensive care admission, separated by whether the pancreatitis was the leading reason for admission

The 9 pancreatitis-driven intensive care admissions were distributed very unevenly across the 16 years: one each in 2011 and 2014, then none for nine consecutive years, then three in 2024 and four in 2025 (Fig. 4). Under a uniform distribution across the 16 years, 1.1 such admissions would have been expected in a two-year window (probability of seven or more 0.0002), and Poisson regression showed an increase of 30% per calendar year (rate ratio 1.30, 95% CI 1.06–1.59; *P* = 0.011). This clustering was not accompanied by a corresponding change of the number of patients treated. 10 patients presented in 2024–2025 and 49 in the preceding 14 years, that is 5.0 versus 3.5 patients per year, a difference well within the year-to-year fluctuation of the whole period and without an upward trend in the same model (rate ratio 1.00 per calendar year, 95% CI 0.95–1.06; *P* = 0.94; Fig. 3). Neither did the severity distribution change: severe disease was graded in 1 of the 10 patients of 2024–2025 and in 4 of the 49 patients before, and the proportion of mild episodes was likewise unchanged. Over 16 years our institution therefore treated a stable number of children with a stable severity distribution, while the proportion of them who received intensive care rose markedly in the final two years.

### The intensive care patients of 2024 and 2025

The eight children treated at our PICU between May 2024 and November 2025 are summarized in Table 1. Two courses illustrate the range of disease and are described in detail. 

#### A fatal course in severe obesity

 A 13-year-old boy with a BMI of 47 kg/m² (> 99th percentile) was transferred by helicopter with SIRS, hypovolemic shock and acute kidney injury. Laboratory findings showed extreme leukocytosis (41.6 × 10⁹/L) and a CRP of 342 mg/L within the first 72 h, cholestasis and an early phase of disseminated intravascular coagulation; serum lipase was 24 times the ULN. MRCP demonstrated extensive necrotizing pancreatitis of head, body and tail with large peripancreatic collections. ERCP identified prepapillary gallstones with distal biliary edema. Because the coagulation was deranged at that time (prothrombin time 58% of normal, INR 1.3), a papillotomy was deliberately not performed and a biliary stent was placed without sphincterotomy and stone extraction. After an intermittent phase of stabilization the patient became septic on the thirteenth treatment day. A repeat procedure showed the stent to be obstructed; it was removed, papillotomy was performed and multiple gallstones were extracted. The course progressed to infected pancreatic necrosis, septic shock and multiorgan failure, and the patient died 17 days after admission. *Enterobacter cloacae* and *Enterococcus faecium* were identified in blood cultures as well as in pancreatic tissue in post-mortem analyses. Genetic testing for hereditary pancreatitis revealed no pathogenic variants.

#### Necrosis with successful endoscopic drainage

 A 16-year-old boy (BMI 17 kg/m², 5th percentile) was referred with severe belt-like abdominal pain and repeated bilious vomiting, lipase on admission was 32 times the ULN. Contrast-enhanced CT confirmed acute necrotizing pancreatitis with extensive parenchymal loss of the pancreatic body and large acute necrotic collections extending retroperitoneally, which evolved into a partially encapsulated collection. Given clinical signs of SIRS, intravenous antibiotic therapy with imipenem/cilastatin was initiated; circulatory parameters remained stable throughout and catecholamines were never required, so that the episode was graded as moderately severe despite the extent of the necrosis. Endoscopic ultrasound initially revealed an immature necrotic collection with solid components, and drainage was deferred. After capsule maturation, transgastric drainage with a lumen-apposing metal stent (15/10 mm) followed by double-pigtail prostheses was successfully performed; the stent was removed after six weeks. Genetic testing identified two variants in the CTRC gene. One month later the patient was re-admitted with a recurrent episode without evidence of infection, which resolved under conservative management, and the disease has since been classified as CP.

### Sensitivity analyses

Two included patients had an enzyme elevation below the three-fold threshold (1.9 and 2.97 times the ULN) and were included after adjudication, one of them on the basis of a sonographic finding, the other one on the clinical constellation documented by the treating team. Repeating the evaluation without them (*n* = 57) changed no proportion by more than 2.9% points, the largest shift being the share of IBD-associated cases (16.9% to 14.0%). Conversely, 2 excluded patients had an enzyme elevation between two and three times the ULN with a compatible presentation but unremarkable imaging; adding them (*n* = 61) changed no proportion by more than 1.5% points. In neither direction did the number of patients requiring intensive care change, since none of the four patients concerned was treated at an intensive care unit.

## Discussion

The observation that led to this study—seven children requiring intensive care for pancreatitis within 19 months at a center that had seen two such patients in the preceding 14 years—is real, but our analysis of the complete 16-year series does not support the interpretation that seemed most plausible at the bedside. Over the same period the number of children treated for pancreatitis at our institution did not change, and neither did the distribution of disease severity. The clustering in our single-center analysis is therefore rather a change of the intensity of care delivered and not a demonstrable change of the disease.

Three explanations have to be weighed. The first is a genuine increase of severe disease. Our data do not support it: severe disease according to the NASPGHAN criteria was graded in 1 of 10 patients in 2024–2025 and in 4 of 49 patients before, and the proportion of mild episodes was unchanged as well. Two of the seven pancreatitis-driven intensive care admissions of 2024–2025 concerned patients with mild disease, one of them was admitted for refractory pain and not for organ dysfunction. Severe courses were moreover present throughout the observation period and not only at its end. In 2011 a 17-year-old girl with obesity and a metabolic syndrome developed necrotizing pancreatitis with a pseudocyst, SIRS, pleural effusion and ascites and spent 25 days in hospital under intensive care—a very similar phenotype as the fatal case of 2024, 13 years earlier.

The second explanation is a concentration of complicated cases by referral, supported by a documented change of local capability. In 2018 a child of our cohort requiring therapeutic ERCP had to be transferred to another university center because pediatric interventional pancreaticobiliary endoscopy was not routinely available here. In the following years, four such procedures—three ERCP with papillotomy and stenting and one endoscopic ultrasound-guided transgastric drainage—were performed in our own house through a structured cooperation starting 2020 between pediatric intensive care, pediatric gastroenterology and adult interventional gastroenterology. A center that acquires the ability to treat complicated pancreatitis is likely to begin receiving complicated pancreatitis.

The third explanation is a lower threshold for intensive care admission once this capability and the corresponding awareness are in place. The girl with mild SPINK1-associated recurrent pancreatitis who was admitted to the PICU for opioid analgesia illustrates this directly; it is likely that the same child would have been managed at a general ward a decade earlier.

These explanations are not mutually exclusive and we cannot quantify their contributions. With nine intensive care admissions over 16 years the estimate is imprecise, and the exploratory character of this analysis has to be kept in mind. The interpretation which came most naturally to us at the bedside—that pediatric pancreatitis in our region had become more dangerous—is the one explanation our own data actively fail to support. A single institution is not an adequate basis for inferring a change of incidence or severity [1, 17], and our analysis illustrates concretely how such an impression arises and how it can be tested.

Biliary disease and IBD were the most frequent etiologies in our cohort with 10 patients each, which is in line with published pediatric series [18]. 7/10 of our patients with biliary pancreatitis were obese or overweight, and the one death of the cohort occurred in an adolescent with a BMI of 47 kg/m². Obesity is an established risk factor for gallstone disease and gallstones are an established cause of pediatric AP [19], and childhood obesity has quadrupled within four decades [20]. In our cohort, however, obesity was associated with the biliary etiology and not with severity or with the need for intensive care: it was documented in 13 of 59 patients overall, but in only 2 of the 15 patients treated at an intensive care unit. Published data on the impact of obesity on the course of pediatric AP are not uniform either [21]. Reported rates of pancreatitis in pediatric IBD cohorts of 1.7–5.1% represent a substantially increased risk over the background population [22, 23], and the incidence of pediatric-onset IBD continues to rise [24]. Genetic variants accounted for 7 patients and dominated, as expected, among those with recurrent and chronic disease [25]. That only 4 of 59 patients remained without an explanation is in keeping with the reported decline of the idiopathic category when the diagnostic work-up becomes more systematic [26].

Twelve of our patients did not live in the catchment area, and these were almost exclusively children who fell ill or were injured while spending their holidays at the Baltic coast. Trauma accounted for 7 patients of the whole cohort, which is a considerable proportion, and pediatric centers in tourist regions should expect such a case mix.

Predictive scores for severe disease are established in adults, and pediatric modifications including CRP, hematocrit and blood urea nitrogen perform reasonably well [27–29]. Our cohort is too small to evaluate or to propose risk prediction, and the two courses described above are presented as illustrations of recognized warning features and not as evidence for a prediction rule. The fatal case emphasizes the importance of close monitoring of children with extensive necrosis for early detection of sepsis [30].

The interventional requirements of these children underline the need for structured interdisciplinary cooperation. Success rates of pediatric ERCP exceed 90% in retrospective series with reported complication rates of 4–12% [31, 32], but the number of pediatric cases at a single center is small and structured training for pediatric endoscopists is still rare, so that most interventions depend on expertise from adult gastroenterology [33]. The cooperation between pediatric intensive care, pediatric gastroenterology and interventional endoscopy recommended by ESPGHAN and NASPGHAN describes exactly the arrangement that made the in-house management of our recent cases possible [34].

Finally, children discharged after an episode of AP need a regular follow-up. 9 of our 59 patients had more than one documented episode and 8 met the criteria for CP. Genetic mutations are a major risk factor for progression to recurrent disease and obesity aggravates it [35]; exocrine insufficiency seems to be prolonged in more than half of children after AP [36], and pre-diabetes and diabetes show a high prevalence in children with genetic mutations or severe AP [37]. About half of the children report a decreased quality of life one year after the first episode [38].

### Limitations

This is a retrospective single-center study, and its main limitations follow from that design. Severity was graded retrospectively from the discharge documentation, which has become more detailed over the observation period, so that severity and intensive care use in the early period may be systematically under-recorded. This bias could by itself produce an apparent recent increase of intensive care use, although it is unlikely to explain a nine-year interval without a single pancreatitis-driven intensive care admission. Case identification rested on discharge coding, and a child whose pancreatitis was never coded would not have been retrieved; a complementary search of the laboratory system for elevated amylase and lipase values would be the logical next step. In one patient no documentation could be retrieved at all. The reference interval for serum lipase changed twice during the study period, so that values close to the three-fold threshold carry some uncertainty; the sensitivity analyses address the patients concerned.

## Conclusions

Pediatric pancreatitis at our tertiary center did neither become more frequent nor more severe over 16 years, but the proportion of affected children who received intensive care rose markedly and concentrated in the last two years of the observation period. The most plausible explanations are a concentration of complicated cases by referral once advanced pediatric pancreaticobiliary endoscopy became available locally, and a correspondingly lower threshold for intensive care admission, although the number of events is small and the estimate correspondingly imprecise. The clinical spectrum was broad, ranging from mild self-limiting episodes to fatal necrotizing pancreatitis, and biliary disease, IBD, trauma and genetic predisposition accounted for the majority of cases. However, the perceived local increase of severe pediatric pancreatitis was not explained by a a change in the spectrum of causes of the disease.

## Data Availability

The datasets used and/or analysed during the current study are available from the corresponding author on reasonable request.

## Abbreviations

AP: Acute pancreatitis
ARP: Acute recurrent pancreatitis
BMI: Body mass index
CP: Chronic pancreatitis
CRP: C-reactive protein
CT: Computed tomography
CTRC: Chymotrypsin C
ERCP: Endoscopic retrograde cholangiopancreatography
ESPGHAN: European Society for Paediatric Gastroenterology Hepatology and Nutrition
EUS: Endoscopic ultrasound
IBD: Inflammatory bowel disease
ICD-10: International Classification of Diseases 10th revision
INR: International normalized ratio
INSPPIRE: International Study Group of Pediatric Pancreatitis: In Search for a Cure
IQR: Interquartile range
MRCP: Magnetic resonance cholangiopancreatography
NASPGHAN: North American Society for Pediatric Gastroenterology, Hepatology and Nutrition
PICU: Pediatric intensive care unit
SIRS: Systemic inflammatory response syndrome
SPINK1: Serine protease inhibitor Kazal type 1
ULN: Upper limit of normal
